# Pharmacokinetic Characterization of the Gastrointestinal Acute Radiation Syndrome Mitigator YK-4-250 in Male and Female Mice Under Non-Irradiated and Partial Body Irradiation Conditions

**DOI:** 10.21203/rs.3.rs-10022093/v1

**Published:** 2026-07-06

**Authors:** Julius O. Nyalwidhe, Vidya P. Kumar, Shimin Chen, Janette Lockett, Yali Kong, Sanchita P. Ghosh, David Mu, Milton L. Brown

**Affiliations:** Macon & Joan Brock Virginia Health Sciences at Old Dominion University; Uniformed Services University of the Health Sciences; Macon & Joan Brock Virginia Health Sciences at Old Dominion University; Macon & Joan Brock Virginia Health Sciences at Old Dominion University; Macon & Joan Brock Virginia Health Sciences at Old Dominion University; Uniformed Services University of the Health Sciences; Macon & Joan Brock Virginia Health Sciences at Old Dominion University; Macon & Joan Brock Virginia Health Sciences at Old Dominion University

## Abstract

The therapeutic evaluation of novel small-molecule candidates requires a comprehensive understanding of their pharmacokinetic (PK) profiles under both baseline physiological conditions and pathophysiological stress states, such as radiation exposure. This study evaluates YK-4-250, a long acting Tempol-conjugated angiotensin receptor blocker (TCARB) being developed to treat gastrointestinal acute radiation syndrome (GI-ARS). Specifically, the study characterizes YK-4-250's non-compartmental PK profile following oral administration of 20 and 40 mg/kg doses to male and female, non-irradiated and irradiated C57BL/6 mice. Male and female mice were stratified into non-irradiated and partial-body irradiation (PBI) at the LD_50/30_ (14.6 Gy). Serial plasma concentrations were analyzed over a 72-hour timeline using non-compartmental analysis (NCA) to determine key parameters (C_max_, T_max_, AUC, t_1/2_, CL, and Vz).

In the non-irradiated state, oral absorption was rapid, with an identical time-to-peak (T_max_ = 1.0 hr) across all cohorts. Dose escalation from 20 to 40 mg/kg in non-irradiated mice yielded a supra-linear expansion in AUC_last_ (3.76 to 12.53 μg∙hr/mL in males and 6.45 to 18.99 μg∙hr/mL in females), indicating saturation of baseline clearance pathways. Exposure to radiation fundamentally altered these kinetic profiles. At the 20 mg/kg dose threshold post-irradiation, a significant sex-based divergence emerged with irradiated females exhibiting significantly higher and prolonged exposure than irradiated males This resulted in doubling total projected exposure (AUC_inf_ = 8.80 vs.4.37 μg∙hr/mL) due to a 50.1% reduction in systemic clearance and an 85.2% prolongation of elimination half-life (t_1/2_ = 7.81 vs. 3.12 hr). However, at 40 mg/kg of YK-4-250, irradiation acted as a “physiological equalizer,” eliminating the exposure gaps. Total exposure (AUC_inf_ approximately 22 μg∙hr/mL) and clearance converged to nearly equivalent values between the males and females. Concurrently, both sexes experienced a significant, extension of terminal half-life (22.51 hr in males and 18.75 hr in females). The primary differentiating features at 40 mg/kg under radiation were a significant, sex-specific delay in female absorption (T_max_ doubled from 3.0 hr in males to 6.0 hr in females) and a 19.2% higher apparent volume of distribution (Vz) in males. Altogether, these findings suggest that radiation exposure can compromise systemic clearance mechanisms in both male and female mice treated with YK-4-250, potentially via the downregulation of hepatic phase I/II metabolic pathways and altered gastrointestinal transit, which may necessitate precise adaptive dosing regimens for radiation-mitigative or protective indications in higher species.

## 1. Introduction

The successful translation of experimental therapeutics from preclinical discovery to targeted clinical applications hinges upon a granular delineation of their *in vivo* pharmacokinetic (PK) behavior. Characterizing a drug's PK profile under baseline physiological conditions provides an indispensable foundation for drug development. While our research has previously focused on this area,^,^ it is equally critical to evaluate these parameters within specific disease or injury microenvironments.

Pathophysiological perturbations, such as acute radiation syndrome (ARS), radiation-induced systemic inflammation, or therapeutic oncology radiation regimens, frequently disrupt molecular pathways and tissue homeostasis. These perturbations carry the potential to drastically modify gastrointestinal absorption dynamics, alter plasma protein binding fractions, shift systemic volumes of distribution, and suppress functional hepatic metabolic capacities. Consequently, evaluating how these radiation-induced systemic alterations affect the *in vivo* behavior of novel therapeutic candidates is a critical prerequisite for effective countermeasure development.

YK-4-250, a TCARB, is a promising orally available radiation mitigator of Gastrointestinal Acute Radiation Syndrome (GI-ARS) that promotes overall survival following partial body irradiation (PBI). While its pharmacological efficacy has been robustly demonstrated in murine models of radiation mitigation and protection, its pharmacokinetic disposition within non-irradiated versus irradiated microenvironments remains completely uncharacterized. This knowledge gap poses a challenge for clinical translation, as radiation exposure is well-documented to systematically disrupt the very biological systems responsible for drug disposition.^,^

Mechanistically, acute exposure to radiation induces severe systemic oxidative stress^,^ and triggers a cascade of pro-inflammatory cytokines, including IL-1, IL-6, and TNF-α. ^,,,,^ Within the gastrointestinal tract, this inflammatory cascade and direct radiolytic damage compromise intestinal epithelial architectures^,,^ through villus blunting, crypt apoptosis, and the rapid disruption of tight junctions, resulting in severe barrier dysfunction and altered gut motility. Beyond the gut, circulating inflammatory cytokines act as potent transcriptional repressors in the liver, where they can selectively downregulate major functional phase I cytochrome P450 (CYP450) enzymatic expressions and modulate critical phase II drug-metabolizing enzymes and cellular transporter behaviors. Consequently, an individual's capacity to absorb, metabolize, and clear therapeutics can be fundamentally altered in the hours and days following radiation exposure.

There is increasing evidence that sexual dimorphism can influence a patient’s response to radiation,^,^ as well as drug metabolism and PK.^,^ Critically, drug PK is a primary determinant of observed gender differences in the incidence and severity of adverse drug reactions (ADRs). This was starkly demonstrated in a retrospective study examining 86 separate drugs, a staggerig 88 % of these drugs resulted in higher drug exposure (area-under-the-curve) and maximum plasma concentrations in female subjects than in male subjects. Furthermoe, 96 % of the drugs that showed this increased exposure in females also induced a higher incidence of adverse events in women. Based on these recent findings, gender differences in PK may necessitate precise adaptive dosing adjustments for clinical populations.

The molecular mechanisms underlying sex-based physiological differences remain poorly understood. Consequently, most precision medicine strategies rely primarily on mutational or genetic profiling to determine therapeutic interventions, often failing to account for how an individual’s sex can significantly influence drug efficacy. Therefore, it may be vital to map the comparative PK of drugs across both non-irradiated and irradiated states in males and females. Establishing this physiological baseline could be essential for optimizing dose-selection paradigms, enhancing safety margins, and accurately defining the compound's therapeutic window in higher species.

**Figure 1:**
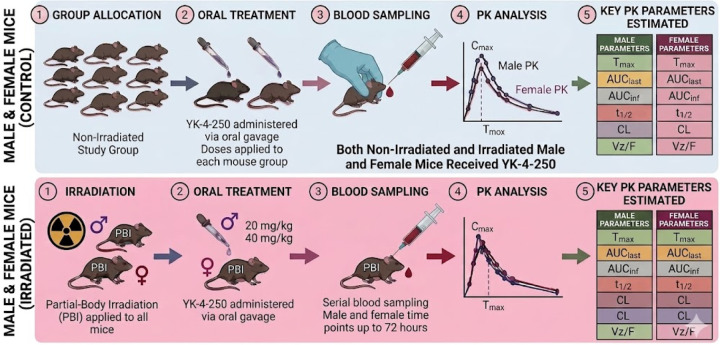
Non-compartmental pharmacokinetics of YK-4-250 in non-irradiated and irradiated mice

Figure 1 illustrates the sequential experimental design utilized to evaluate the YK-4-250's PK profile following radiation exposure. The process initiates with IR, where mice undergo targeted exposure to a 14.6 Gy PBI which represents the LD50/30 in our system. The non-irradiated and irradiated animals are administered the investigational compound YK-4-250 via oral gavage at doses of either 20 mg/kg or 40 mg/kg. Following administration, serial blood collection at specific post-dose time points up to 72 hours were performed. Plasma concentration-time profile graphs were constructed to map the YK-4-250's absorption and elimination trends. Finally, the resulting non-compartmental PK metrics were calculated from the plasma curves, explicitly defining parameters (C_max_, T_max_, AUC_last_ and AUC_inf_, t1/2, CL, and apparent volume of distribution (Vz/F)) for males and females. Ultimately, by integrating these sex-based and radiation-dependent PK assessments, this research aims to move beyond a “one-size-fits-all” approach, providing a robust framework for evidence-based dose optimization that ensures both the safety and therapeutic efficacy of YK-4-250 in diverse clinical populations.

## 2. Materials and Methods

### 2.1. Synthesis and Preparation of YK-4-250

All chemicals and solvents were purchased from commercial suppliers and used as received unless noted otherwise. The synthesis of YK-4-250 was completed as previously reported.^7^ YK-4-250 was purified by flash chromatography. Liquid chromatography/mass spectrometry (LC/MS) analyses were conducted using Shimadzu LC-20AD pumps and an SPD-20A UV-vis detector. ^1^H-NMR and high-resolution mass spectra (HRMS) were recorded for structural validation. YK-4-250 (20 and 40 mg/kg doses) were prepared for oral gavage as a suspension in a formulation of 5% DMSO and 0.5% Tween 80 in sterile water.

### 2.2. Animal and Veterinary Care

All procedures involving animals were reviewed and approved by the Armed Forces Radiobiology Research Institute’s (AFRRI) Institutional Animal Care and Use Committee (IACUC) using the principles outlined in the National Research Council’s Guide for the Care and Use of Laboratory Animals and in accordance with relevant regulations and guidelines. Mice that showed an inability to remain upright, were unresponsive or showed decreased or labored respiration were considered moribund and euthanized according to the American Veterinary Medical Association (AVMA) guidelines. Animal studies were conducted in compliance with Animal Research: Reporting of *In Vivo* Experiments (ARRIVE) guidelines.

Male and female C57BL/6 mice (12–14 weeks) were purchased from Jackson Laboratories (Bar Harbor, ME). Animals were housed in the AFRRI’s vivarium in plastic cages in Allentown NexGen cage systems. The animals were provided with Harlan Teklad Rodent Diet 8604 (Envigo) and acidified water (pH 2.5–3.0). The cages and the room were kept at a temperature of 20–26°C, humidity between 30–70%, and a 12:12 hour light : dark cycle. Veterinary care was available throughout the study and animals were examined by research and veterinary staff for clinical signs or changes in appearance. The animals were administered either drug or vehicle by oral gavage (PO) as a 1-dose regimen (post-PBI). Mice were euthanized by CO_2_ inhalation plus confirmatory cervical dislocation by investigator/technician according to the directives of the vivarium’s Standard Operating Procedure (Rodent Euthanasia Guidelines) and in accordance with current AVMA Guidelines on Euthanasia

### 2.3. BM2.5-PBI and Dosimetry

Mice for each treatment were placed under anesthesia (3% isoflurane, 97% O_2_) then irradiated using a 4 MV photon beam from an Elektra Infinity clinical linear accelerator (LINAC) using the BM2.5-PBI model as previously described^7,^ to achieve 2. % bone marrow sparing, one hind leg was excluded from the radiation field. Prior to irradiation, dosimetry was confirmed and the beam output was verified through ion chamber measurements (PTW, model 30013). Animals were irradiated at the most recent LD_50/30_ dose (14.6 Gy) at an estimated dose rate of 2.8 Gy/min. Post irradiation, mice were returned to the vivarium and closely monitored for their health status throughout the course of the study. Each study included mice that received vehicle post-irradiation as a control group. Blood drawings were done at times 0, 1, 3, 6, 12, 24, 48 and 72 hrs. Blood samples were labeled and stored at −80 °C until evaluation.

### 2.4. Development and Validation of the YK-4-250 Plasma Calibration Curve and Quantitative LC-MS Analysis

1. **2.4.1. Chemicals and Reagents**. Purified YK-4-250 was obtained as described in Section 2.1. Nimodipine, used as the internal standard (IS), was purchased from Sigma-Aldrich Millipore (Cat. No. PHR1293–16; purity 99%). Mouse plasma (Innovative Grade Mouse C57BL/6) used for calibration curve and quality control (QC) experiments was obtained from Innovative Research (Novi, MI, USA). HPLC-grade methanol, formic acid, and water were purchased from Thermo Fisher Scientific (Waltham, MA, USA). Stock solutions of YK-4-250 and nimodipine were prepared in methanol and stored at −20°C protected from light. Working standard solutions were prepared by serial dilution of stock solutions in methanol to generate calibration standards spanning the analytical range. The identity and purity of YK-4-250 and nimodipine were initially confirmed by high-resolution mass spectrometry (HRMS) using a Thermo Scientific Orbitrap Fusion Lumos mass spectrometer.
2. **2.4.2. Preparation of Calibration Standards and Plasma Samples**. Calibration standards were prepared by spiking 100 μL of mouse plasma with YK-4-250 working standards and 50 μL of concentration normalized nimodipine internal standard solution. Protein precipitation was performed by adding 300 μL of methanol followed by vortex mixing for 1.5 min. Samples were centrifuged at 13,400 rpm for 10 min at 4°C, and the resulting supernatants were transferred to clean microcentrifuge tubes. The extracts were evaporated to dryness under a gentle stream of nitrogen and reconstituted in 100 μL of 0.1% formic acid in water. Reconstituted samples were transferred to autosampler vials and stored at −80°C until LC-MS analysis.
3. **2.4.3. LC-MS Analysis**. Quantitative analysis was performed using a Thermo Scientific Q Exactive Orbitrap mass spectrometer coupled to a Vanquish Flex UHPLC system (Thermo Scientific). A 2 μL aliquot of each sample was injected for MS1 full scan analysis. To minimize analytical bias, the acquisition sequence for approximately 330 processed plasma samples representing all experimental groups was randomized prior to LC-MS analysis. All calibration standards and biological plasma samples were analyzed in triplicate injections. To reduce potential carryover, two blank injections consisting of 0.1% formic acid were performed after triplicate injections. No endogenous interferences were observed in blank mouse plasma under the optimized chromatographic conditions. The retention times of YK-4-250 and nimodipine were approximately 9.2 min and 5.1 min, respectively.
4. **2.4.4. Calibration Curve Construction and Quantification**. The plasma calibration curve was generated using YK-4-250-spiked plasma standards over a concentration range of 0.02–18.0 μg/mL. Calibration was performed by plotting the natural logarithm of the peak-area ratio of YK-4-250 to the internal standard (IS) against the corresponding natural logarithm of analyte concentration. Linear regression analysis yielded the calibration equation: y = 1.27 + 1.51x (R^2^ = 0.99) which was subsequently used to determine YK-4-250 concentrations in plasma samples collected from *in vivo* PK studies (See supplemental Figure S1).
5. **2.4.5. PK Analysis**. Quantified plasma concentrations of YK-4-250 were used for NCA analysis. PK parameters including C_max_, T_max_, AUC, terminal elimination t_1/2_, MRT, apparent CL, and apparent Vz were calculated using standard non-compartmental analysis (NCA) methods. Detailed LC/MS conditions, mass spectrometry acquisition parameters, and dilution schemes and calibration curve are provided in the supplementary materials.

### 2.5. Characterization of Experimental Cohorts

The experimental framework utilized adult male and female mice divided into eight distinct evaluation cohort as provided in Table 1.

### 2.6. Mathematical Non-Compartmental PK Definitions

NCA is a foundational method used to characterize PK profiles directly from observed plasma concentration-time data without assuming a specific multi-compartment structural model. Utilizing NCA, several key metrics were derived from the plasma concentration-time profile to evaluate a YK-4-250's behavior *in vivo*. Peak systemic exposure is defined by the maximum observed C_max_, while the rate of drug absorption or systemic appearance is reflected by the T_max_, which represents the specific time point at which C_max_ occurs. Total observed systemic exposure over the monitored time frame is quantified using AUC_last_, the area under the plasma concentration-time curve calculated from time zero to the last measurable concentration. To capture estimated total drug exposure over an infinite timeline, AUC_inf_ is utilized, which extrapolates the curve to infinity by adding the ratio of the last measurable concentration to the terminal elimination rate constant C_last_/λ_z_. The mathematical reliability of this extrapolation is evaluated by the AUC extrapolated % which measures the percentage of AUC_inf_ contributed by the extrapolated phase, where disproportionately large percentages signal reduced reliability of the absolute AUC_inf_ calculation. Finally, YK-4-250's persistence and duration in the body are captured by the terminal elimination t_1/2_ and the Mean Residence Time (MRT). The t_1/2_ represents the time required for the plasma concentration to decrease by 5 % during the terminal log-linear elimination phase, calculated as ln(2)/λ_z_, whereas the MRT indicates the average lifetime of a drug molecule within the biological system, calculated from the ratio of the area under the first moment curve to the area under the curve (AUMC/AUC).

## 3. Results

### 3.1. Mean Plasma Concentration Data Tables

The specific time-course plasma concentration profiles for each group are recorded directly from the experimental datasets in Tables 2 and 3. The time-course plasma concentration profiles for each group are recorded directly from the experimental datasets, demonstrating distinct disposition patterns based on sex, dose, and radiation exposure. As compiled in the tracking matrix for male groups (Table 2), the non-irradiated animals given 20 mg/kg (PBI-035-2) exhibit a rapid mean plasma absorption peak of 1.1414 μg/mL at 1 hour, which temporarily declines at 3 hours to 0.374 mg/mL, spikes slightly to 0.7438 μg/mL at 6 hours, and subsequently clears below the limit of quantification from 12 hours through the remaining 72-hour timeline. Doubling the dose in non-irradiated males to 40 mg/kg (PBI-035-3) results in a smoother, more sustained clearance profile, peaking at 1.3994 μg/mL at 1 hour before progressively descending through 0.8988 μg/mL at 3 hours, 0.5838 μg/mL at 6 hours, 0.5675 μg/mL at 12 hours, and a final measurable concentration of 0.0748 μg/mL at 24 hours. Conversely, PBI markedly alters male absorption kinetics; the 20 mg/kg irradiated males (PBI-037-2) display a delayed peak plasma appearance of 0.7981 μg/mL at 3 hours, clearing to 0.2284 μg/mL at 6 hours and 0.0962 μg/mL at 12 hours, while the 40 mg/kg irradiated males (PBI-037-3) achieve a much higher delayed peak of 1.8951 μg/mL at 3 hours, with prolonged systemic retention maintaining measurable plasma values of 0.1903 μg/mL, 0.0514 μg/mL, and 0.0434 μg/mL at the 24, 48, and 72-hour intervals, respectively.

Parallel dynamics are captured in the concentration tracking matrix for female groups (Table 3), where baseline and radiation stress factors interact uniquely across both dosing regimens. Non-irradiated female mice administered 20 mg/kg (PBI-036-2) absorb the compound rapidly to reach a peak mean concentration of 1.2903 μg/mL at 1 hour, clearing steadily to 1.1661 μg/mL at 3 hours, 0.2307 μg/mL at 6 hours, 0.0899 μg/mL at 12 hours, and a terminal baseline of 0.0668 μg/mL at 24 hours. Escalating the dose to 40 mg/kg in non-irradiated females (PBI-036-3) increases the 1-hour peak concentration to 2.0026 μg/mL, followed by a minor drop to 1.3051 μg/mL at 3 hours, a secondary elevation to 1.6046 μg/mL at 6 hours, and extended quantifiable elimination levels of 0.3433 μg/mL at 12 hours, 0.1081 μg/mL at 24 hours, and 0.0393 μg/mL at 48 hours. When exposed to acute PBI, the 20 mg/kg female cohort (PBI-038-2) maintains a rapid 1-hour absorption peak of 0.911 μg/mL, falls to 0.4388 μg/mL at 3 hours, climbs to a secondary peak of 0.6866 μg/mL at 6 hours, and registers to 0.2278 μg/mL at 12 hours before dropping to zero by 24 hours. Finally, the combination of irradiation and the 40 mg/kg dose threshold in females (PBI-038-3) causes a profound delay in absorption influx alongside significant excretory blockade. This cohort tracks initial mean values of 1.1089 μg/mL at 1 hour and 0.7956 μg/mL at 3 hours before achieving a late peak plasma concentration of 1.6815 μg/mL at 6 hours, which then clears slowly to 0.7461 μg/mL at 12 hours, 0.1292 μg/mL at 24 hours, 0.0652 μg/mL at 48 hours, and remains detectable at 0.0219 μg/mL at the final 72-hour collection point.

### 3.2. Comparative PK Analysis by Sex and Dose

#### 3.2.1 Comparative Pharmacokinetic Evaluation under Non-Irradiated States

Non-compartmental pharmacokinetic analysis presented in Table 4 reveals profound sex-based discrepancies in the systemic disposition of YK-4-250 in irradiated mice. The baseline pharmacokinetic profile at the 20 mg/kg dose in non-irradiated control mice reveals stark mathematical anomalies between the sexes, driven almost entirely by poor terminal phase capture in the male cohort. At the beginning of the kinetic profile, absorption appears relatively uniform; both male and female mice share an identical time-to-peak concentration (T_max_) of 1 hour, with females exhibiting a modest 13.05% higher peak concentration (C_max_) of 1.2903 μg/mL compared to 1.1414 μg/mL in males. This trend of higher female exposure persists through the actual measured time points, with females displaying a substantial 71.51% higher AUC_last_ (6.4536 μg·hr/mL) than males (3.7628 μg·hr/mL). However, the data diverges into biologically unrealistic territory when looking at total extrapolated exposure (AUC_inf_), where females suddenly appear to have a 52.98% lower total exposure than males.

This paradox is explained by the massive 79.48% extrapolated AUC (AUC_extra_) in the male group. In standard pharmacokinetics, any extrapolation exceeding 20% indicates that the blood sampling did not continue long enough to capture the true terminal elimination phase. Because nearly 80% of the male AUC_inf_ is based on mathematical projection rather than actual data, it skews all downstream parameters for the male mouse. This mathematical artifact causes a severe underestimation of male clearance, resulting in a physically implausible 1712.43% apparent increase in female clearance (CL) and a corresponding 106.70% inflation of the female apparent volume of distribution (Vz). Interestingly, despite these heavy distortions in clearance and distribution calculations, the calculated terminal half-lives (t_1/2_) managed to remain virtually identical, with females showing a minor 2.82% decrease (11.0805 hours) compared to males (11.4017 hours). Ultimately, while the raw AUC_last_ confirms that females experience higher initial systemic exposure at 20 mg, any definitive conclusions regarding baseline sex differences in clearance and distribution at this dose must be heavily qualified due to the unreliable terminal data in the male cohort.

The pharmacokinetic profile of the 40 mg/kg dose in non-irradiated control mice reveals a profound and statistically significant divergence based on biological sex, with female mice demonstrating markedly higher systemic exposure and prolonged drug retention compared to males (Table 5). Peak systemic concentration (C_max_) is 43.10% higher in females (2.0026 mg/mL) than in males (1.3994 mg/mL), despite an identical time-to-peak absorption (T_max_) of 1 hour for both groups. This immediate difference in blood concentration translates into a substantially higher overall drug exposure over time, with females exhibiting a 51.60% increase in AUC_last_ and a 49.71% increase in total projected exposure (AUC_inf_).

This increased exposure in female mice is primarily driven by a 33.21% reduction in systemic clearance (CL) compared to males (2.0329 versus 3.0435 mg/μg·hr/mL), indicating that females clear the compound at a much slower rate. Consequently, the terminal elimination half-life (t1/2) in females is more than double that of males, representing a dramatic 111.65% increase (12.0281 hours in females versus 5.6830 hours in males). Interestingly, females also exhibit a 41.37% increase in the apparent volume of distribution (Vz), suggesting greater tissue distribution. Typically, a larger Vz can extend half-life by keeping the drug distributed in peripheral tissues rather than in the blood where it can be cleared. In this case, the combination of a broader tissue distribution and a significantly restricted clearance rate forces YK-4-250 to reside in the female system for a vastly extended duration compared to their male counterparts under non-irradiated conditions.

### 3.3 Pathophysiological Kinetic Alterations under IR

The induction of the LD_50/30_ PBI dramatically transforms the physiological landscape of drug disposition, profoundly altering the toxicokinetic behavior of YK-4-250 and masking normal baseline sex differences. At the 20 mg/kg dose threshold under IR, a distinct split emerges between the sexes (Table 6). Following exposure to radiation, the 20 mg/kg PK profile demonstrates distinct, sex-dependent alterations, characterized by significantly accelerated drug absorption and highly prolonged systemic retention in female mice compared to males. Unlike the non-irradiated controls where absorption timing was uniform, irradiation induces a stark divergence in YK-4-250 entry. Female mice reach peak concentration three times faster than males, exhibiting a 66.67% decrease in T_max_ (1 hour in females versus 3 hours in males). While the initial peak concentration (C_max_) is only moderately higher in females by 14.15%, the combination of faster absorption and drastically impaired elimination leads to a major accumulation of the drug over time. This is clearly reflected in the exposure metrics, where females display a 58.17% higher AUC_last_ and a striking 101.37% increase in total projected exposure (AUC_inf_), effectively doubling the overall drug burden seen in the male cohort.

This pronounced increase in total female exposure is directly tied to a severe reduction in their ability to clear the compound post-irradiation. Systemic clearance (CL) is halved in female mice, showing a 50.137% decrease (2.3954 mg/μg·hr/mL compared to 4.8040 mg/μg·hr/mL in males). Because the females clear the drug at half the rate of the males, the terminal elimination half-life (t_1/2_) in females stretches by 85.23%, expanding to 7.8141 hours while males eliminate the drug rapidly with a half-life of just 3.1210 hours. Furthermore, females exhibit a 24.85% increase in the apparent volume of distribution (Vz), moving from 21.6306 in males to 27.0048 in females, indicating a moderately broader distribution of the drug into peripheral tissues. Taken together, these findings indicate that at the 20 mg/kg dose, female mice had elevated YK-4-250 exposure and a significantly delayed elimination timeline, driven by an accelerated rate of absorption alongside a heavily suppressed clearance capacity.

### 3.4 The “Radiation Equalizer” Effect at the 40 mg/kg Dose

The true translational significance of this study is revealed at the 40 mg/kg dose under radiation injury, where the severe pathophysiological stress of LD_50/30_ 14.6 Gy PBI acts as a potent physiological equalizer, blunting inherent sex-specific metabolic differences (Table 7). At the YK-4-250 dose of 40 mg/kg under irradiated conditions, the PK profiles of male and female mice show a remarkable convergence in total systemic exposure and clearance, yet they diverge significantly in their absorption rates and tissue distribution kinetics. Female mice exhibit a minor 11.27% lower peak concentration (C_max_) of 1.6815 μg/mL compared to 1.8951 μg/mL in males, but the time required to reach this peak (T_max_) doubles, showing a 100% increase from 3 hours in males to 6 hours in females. This indicates a significant, sex-specific delay in gastrointestinal absorption or physiological uptake for the 40 mg/kg irradiated female group. Despite this distinct delay in peak timing, the total YK-4-250 exposure over time remains remarkably stable between males and females. Females show just a minor 7.20% increase in AUC_last_ and a negligible 3.03% increase in total projected exposure (AUC_inf_).

This stabilization of overall exposure is mathematically mirrored by an almost identical systemic clearance (CL) rate, with females exhibiting a minor, negligible 2.94% decrease compared to males (1.7637 vs. 1.8172 mg/μg·hr/mL). Interestingly, both sexes experience a massive, dramatic increase in terminal half-life (t_1/2_) at this high dose, with the drug lingering for 22.5087 hours in males and 18.7456 hours in females (a 16.72% shorter half-life in females relative to males). Because clearance rates are practically identical, this prolonged retention is heavily influenced by changes in tissue distribution. Males exhibit a 19.17% higher apparent volume of distribution (Vz = 59.0088) than females (Vz = 47.6980), indicating that the drug distributes more extensively into the peripheral tissues of irradiated males, which ultimately drives their slightly longer terminal half-life. Overall, while a 40 mg/kg dose coupled with irradiation forces a profound, universal slowdown in drug elimination for both sexes, it eliminates the massive exposure gaps seen at the 20 mg/kg dose, leaving absorption delay in females and enhanced tissue distribution in males as the primary differentiating features.

### 3.5. Global Noncompartmental PK Parameter Matrix

**Figure 2:**
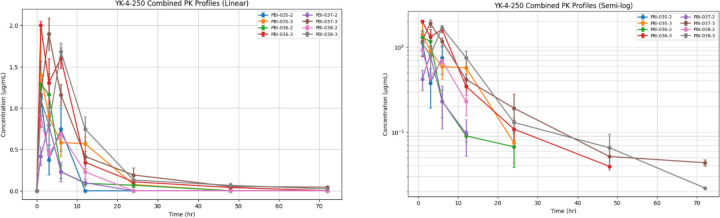
Summary of PK Profile of all Subjects in this Cohort

The summary parameters calculated across all eight subjects are presented in Table 8 and in Fig. 2. Based on the NCA data, the parameters summarized in the consolidated pharmacokinetic matrix provide a granular mapping of how nominal dose, physical radiation stress, and biological sex interact to shape the systemic disposition of YK-4-250.

#### 3.5.1. Absorption Kinetics and Peak Exposure (C_max_ and T_max_)

In non-irradiated animals, YK-4-250 undergoes rapid oral absorption across the intestinal epithelium, achieving a peak plasma T_max_ at exactly 1.0-hour post-gavage across both male and female groups (Table 8). In these baseline states, escalating the oral dose from 20 mg/kg to 40 mg/kg results in a predictable increase in peak C_max_, moving from 1.1414 μg/mL to 1.3994 μg/mL in males, and from 1.2903 μg/mL to 2.0026 μg/mL in females.

*In vivo* tissue irradiation can systematically delay absorption influx velocity. In irradiated males, the T_max_ is delayed from 1.0 hour to 3.0 hours at both dose tiers, though they still achieve substantial peak concentrations (C_max_ of 0.7981 μg/mL at 20 mg/kg and 1.8951 μg/mL at 40 mg/kg). This delay is even more pronounced in the 40 mg/kg irradiated female cohort, where the T_max_ shifts to 6.0 hours post-dose, achieving a late C_max_ of 1.6815 μg/mL. This temporal lag could align with radiation-induced gastric emptying delays and altered structural enterocyte integrity.

#### 3.5.2. Systemic Exposure Parameters (AUC_last_ and AUC_inf_)

In non-irradiated animals, doubling the dose from 20 mg/kg to 40 mg/kg yields a non-proportional, supra-linear expansion in total observed exposure (AUC_last_) for males, rising from 3.7628 to 12.5295 μg/mL (Table 8). In non-irradiated females, a similar dose escalation expands the AUC_last_ from 6.4535 to 18.9944 μg/mL, reflecting potential saturation of baseline clearance pathways at higher concentrations.

Exposure to the LD_50/30_ PBI drives a substantial accumulation in systemic exposure at the higher 40 mg/kg dose. Irradiated males at 40 mg/kg track an elevated AUC_inf_ of 22.0124 μg/mL compared to 13.1428 μg/mL in their non-irradiated counterparts. Similarly, irradiated females treated with a dose of 40 mg/kg demonstrate an elevated AUC_inf_ of 22.6795 μg/mL. This confirms that physical radiation stress significantly boosts total bioavailability or stall clearance mechanisms, sustaining high systemic exposure of the mitigator over an extended timeline.

For the 20 mg/kg non-irradiated male group, the AUC extrapolated % is high (76.4791%), indicating rapid early clearance below the limit of quantification and suggesting that the absolute AUC_inf_ values for the 20 mg/kg dose cohorts should be interpreted with caution. Conversely, all 40 mg/kg treated animals demonstrate tight, highly reliable terminal linear fits with extrapolation percentages under 10%, fulfilling standard regulatory criteria for robust pharmacokinetic analysis.

#### 3.5.3. Elimination and Clearance Efficiencies (t _1/2_ }, MRT, and CL)

Apparent CL values drop markedly under radiation stress (Table 8). Non-irradiated males at 40 mg/kg exhibit a clearance of 3.0435 mg/(μg·hr/mL), which drops down to 1.8172 mg/(μg·hr/mL) in the setting of irradiation. A parallel drop is observed in the female 40 mg/kg mouse, falling from 2.0329 to 1.7637 mg/(μg·hr/mL). This clearance suppression potentially points toward a downregulation or metabolic blockade of hepatic CYP enzymes and transporter pathways induced by circulating radiolytic cytokines.

Reflecting this excretory stall, the terminal elimination t_1/2_ and Mean Residence Time (MRT) are elongated following irradiation. At the 40 mg/kg threshold, the t_1/2_ expands from 5.683 hours in non-irradiated males to 22.5087 hours in irradiated males. In females, the t_1/2_ increases from 12.0281 hours to 18.7456 hours upon irradiation. The MRT follows an identical trend, almost doubling in males (from 8.8574 to 18.9935 hours), emphasizing that the drug resides in an irradiated system far longer than a non-irradiated controls.

#### 3.5.4. Apparent Vz

The apparent volume of distribution during the terminal phase Vz increases substantially in high-dose irradiated states. In the 40 mg/kg male cohorts, Vz expands from 24.9531 to 59.0088 apparent volume units when moving from a non-irradiated to an irradiated state (Table 8). In females at the same dose, it moves from 35.2766 to 47.6980 units. This finding indicates extensive peripheral tissue penetration, altered plasma protein binding fractions, or enhanced vascular permeability/extravasation into damaged systemic microenvironments, such as the injured gastrointestinal tract, where the radiation mitigator YK-4-250 is physically required to exert its therapeutic effects.

## 4. Discussion

### 4.1. Impact of Pathophysiological IR on Absorption Kinetics

Figure 2 provides a summary of all PK profiles in the study. Individual analysis is provided in supplemental figures S2-S9. A profound insight gained from this non-compartmental investigation is the significant lag in gastrointestinal absorption induced by IR exposure. In non-irradiated physiological environments, YK-4-250 rapidly enters systemic circulation, consistently achieving peak plasma profiles within 1.0-hour post-gavage across genders (T_max_ = 1.0 hr). Under irradiated constraints, however, T_max_ is systematically shifted into delayed windows, reaching 3.0 hours in males (PBI-037-2 and PBI-037-3) and up to a late 6.0 hours in the 40 mg/kg females (PBI-038-3). This behavior can be explained mechanically via radiation-induced acute mucosal physiological damage. PBI at the LD_50/30_ (14.6 Gy) compromises the microvascular architecture of the stomach and small intestine,^7^ potentially slowing gastric emptying rates, and limiting smooth-muscle coordinated motility. The prolonged presence of YK-4-250 within the compromised gastrointestinal lumen slows the rate of absorption, resulting in a broadened absorption curve and a delayed T_max_.

### 4.2. Alteration of Hepatic and Systemic Clearance Mechanisms

The 40 mg/kg irradiated groups demonstrated a substantial reduction in clearable efficiency compared to non-irradiated baseline metrics (Table 8). The apparent systemic CL dropped down to 1.8172 in males and 1.7637 μg·hr/mL in females. This severe reduction in clearance matches a profound extension of the terminal biological t_1/2_, which reached 22.51 hours (PBI-037-3) and 18.75 hours (PBI-038-3) for males and females, respectively. This marked drug retention implies that IR suppresses hepatic metabolic clearance pathways. It is well established that radiation-provoked cascades upregulate free radical formation along with circulating inflammatory markers (such as IL-1, IL-6, and TNF-α). These cytokines can act as transcriptional repressors of major functional phase I CYP monooxygenases (such as the CYP3A and CYP2C families) and relevant phase II conjugating transferases within hepatic tissue. With functional enzymatic pools depleted, the metabolic processing of YK-4-250 slows down, leading to elevated systemic accumulation (AUC_last_ values rising to 20.6031 and 22.0872 μg·hr/mL) and prolonged circulatory retention.

### 4.3. Evaluation of Dose Proportionality and Sexual Dimorphism

The compound demonstrates highly complex, non-linear kinetics across the dose ranges evaluated. In non-irradiated male animals, scaling up from 20 to 40 mg/kg yields a disproportionately higher AUC_last_ (increasing from 3.7628 to 12.5295 μg·hr/mL), which could indicate saturation of first-pass hepatic extraction or gut-wall efflux pumps (Table 8). Minor variations based on sex were also observed. In non-irradiated states, female mice demonstrated larger volumes of distribution (Vz = 35.27–42.51) compared to their male counterparts (Vz = 20.56–24.95), suggesting different lean-to-adipose tissue distribution ratios. However, under severe radiation stress, these baseline gender differences were minimized by the overarching systemic metabolic suppression. Both sexes exhibited similar, prolonged clearance limits (CL ≈ 1.8 mg/(μg·hr/mL)), showing that radiation-induced physiological stress overrides typical baseline variations in drug disposition.

## 5. Conclusions

YK-4-250, a long acting Tempol conjugate of Telmisartan, is a novel mitigator of GI-ARS.^7^ Non-compartmental pharmacokinetic analysis yields four major operational findings regarding its *in vivo* systemic behavior. Following irradiation, substantial sex-based differences in pharmacokinetics are observed, though these variations are highly dependent on the administered dose. At the lower dose of 20 mg/kg, irradiated females (Table 8) experience significantly higher and more prolonged systemic drug exposure than irradiated males. Specifically, total projected exposure (AUC_inf_) doubles in females due to a 50.3% reduction (4.3724 vs 8.8047 μg∙hr/mL) in clearance, which subsequently drives a 150.4% prolongation of the elimination half-life (3.121 vs. 7.8141 hr) and a 93.5% increase in mean residence time (5.3498 vs. 10.3541 hr). Interestingly, absorption is faster in females at this lower dose, with peak concentrations (T_max_) achieved at 1 hour compared to 3 hours in males.

However, when the dose is escalated to 40 mg/kg, these metabolic differences largely disappear, suggesting that the physiological pathogenetic pathways or metabolic enzymes causing the sex discrepancy at lower doses become saturated. At 40 mg/kg, total exposure (AUC_inf_), clearance, and half-life converge to nearly equivalent values between the sexes. At the 40 mg/kg dose, the most striking divergence occurs in absorption timing. The female absorption profile becomes severely delayed, with T_max_ doubling from 3 hours in males to 6 hours in females.

Based on experimental findings, dose modifications might be justified and highly recommended when treating irradiated subjects, primarily stratified by biological sex at 20 mg/kg, and by clinical timing at 40 mg/kg doses. At the 20 mg/kg dose, a downward dose adjustment or an extended dosing interval could be considered for irradiated females. Because their total drug exposure is twice that of males and the drug's half-life is vastly prolonged, maintaining a standard male-equivalent dose in females runs a significant risk of accumulation and potential systemic toxicity. Conversely, irradiated males clear the drug rapidly and may require a higher or more frequent dose to maintain therapeutic efficacy.

In regard to the 40 mg/kg dose, absolute daily dose modifications between males and females may not be necessary, as their total systemic exposures (AUC) and clearances are virtually identical. However, while the total amount of YK-4-250 *in vivo* is the same at 40 mg/kg, translational researchers might need to account for the severe absorption delay in females (T_max_ = 6 hours). Since YK-4-250 is intended for acute or immediate therapeutic effect in radiation countermeasures, the delayed peak in females could result in a lag in clinical efficacy. In such scenarios, alternative strategies, such as an initial intravenous loading dose or a split-dose regimen, could be explored for female patients to bypass the irradiation-induced delay in gastrointestinal absorption. Ultimately, evaluating tailored, sex-specific dosing and scheduling strategies in higher species will be important to assess the therapeutic index of YK-4-250, ensuring safe, rapid, and effective mitigation of GI-ARS for both males and females.

In conclusion, this study provides the first systematic characterization of the non-compartmental pharmacokinetics of YK-4-250 under baseline and acute radiation conditions, establishing a vital translational foundation for the medical countermeasure community. Because physical radiation injury systematically disrupts the very biological systems responsible for drug disposition, normal therapeutic dosing regimens developed for uninjured states might not be applied directly to irradiated subjects without risking accumulation-related toxicities.

## Supplementary Material

Supplementary Files

This is a list of supplementary files associated with this preprint. Click to download.

- SupplementalFiguresPKpaper.docx

## Figures and Tables

**Table 1:** Experimental Cohort Design and Dosing Stratification of Mice by Sex and Irradiation Status

Cohort	Sex	Irradiation status	Dose (mg/kg)
PBI-035-2	M	non-irradiated	20
PBI-035-3	M	non-irradiated	40
PBI-036-2	F	non-irradiated	20
PBI-036-3	F	non-irradiated	40
PBI-037-2	M	irradiated	20
PBI-037-3	M	irradiated	40
PBI-038-2	F	irradiated	20
PBI-038-3	F	irradiated	40

**Table 2:** Mean Plasma Concentration Tracking for Male Subjects in this Study (μg/mL ± SEM)

Time (hr)	PBI-035-2 (Non-irradiated 20 mg/kg)	PBI-035-3 (Non-irradiated 40 mg/kg)	PBI-037-2 (Irradiated 20 mg/kg)	PBI-037-3 (Irradiated 40 mg/kg)
**0**	0 ± 0	0 ± 0	0 ± 0	0 ± 0
**1**	1.1414 ± 0.3583	1.3994 ± 0.4864	0.4184 ± 0.1110	1.1657 ± 0.4001
**3**	0.374 ± 0.1823	0.8988 ± 0.1056	0.7981 ± 0.0718	1.8951 ±0.1978
**6**	0.7438 ± 0.2662	0.5838 ± 0.1694	0.2284 ± 0.1199	1.1582 ± 0.1287
**12**	0 ± 0	0.5675 ± 0.0855	0.0962 ± 0.0440	0.4139 ± 0.0666
**24**	0 ± 0	0.0748 ± 0.0320	0 ± 0	0.1903 ± 0.0889
**48**	0 ± 0	0 ± 0	0 ± 0	0.0514 ± 0.0122
**72**	0 ± 0	0 ± 0	0 ± 0	0.0434 ± 0.0038

**Table 3:** Mean Plasma Concentration Tracking for Female Subjects in this Study (μg/mL ± SEM)

Time (hr)	PBI-036-2 (Non-irradiated 20 mg/kg)	PBI-036-3 (Non-irradiated 40 mg/kg)	PBI-038-2 (Irradiated 20 mg/kg)	PBI-038-3 (Irradiated 40 mg/kg)
**0**	0 ± 0	0 ± 0	0 ± 0	0 ± 0
**1**	1.2903 ± 0.1260	2.0026 ± 0.0499	0.911 ± 0.1113	1.1089 ± 0.1521
**3**	1.1661 ± 0.2506	1.3051 ± 0.2942	0.4388 ± 0.0547	0.7956 ± 0.2433
**6**	0.2307 ± 0.0825	1.6046 ± 0.1236	0.6866 ± 0.0446	1.6815 ± 0.1062
**12**	0.0899 ± 0.0135	0.3433 ± 0.0733	0.2278 ± 0.0725	0.7461 ± 0.1507
**24**	0.0668 ± 0.0284	0.1081 ± 0.0280	0 ± 0	0.1292 ± 0.0173
**48**	0 ± 0	0.0393 ± 0	0 ± 0	0.0652 ± 0.0298
**72**	0 ± 0	0 ± 0	0 ± 0	0.0219 ± 0

**Table 4:** Consolidated Non-Compartmental Pharmacokinetic Summary of Non-Irradiated Male and Female C57BL/6 Mice Following Oral Administration of 20 mg/kg of YK-4-250.

PK Parameter	Male Mice (M)	Female Mice (F)	Sex-Based Variance Analysis (% Difference or Shift)
**C_max_** **(μg/mL)**	1.1414	1.2903	Females exhibit an 13.05% higher C_max_
**Tmax (hr)**	1	1	Absorption profiles are identical
**AUC_last_** **(μg·hr/mL)**	3.7628	6.4536	Females exhibit an 71.51% higher AUC_last_
**AUCinf (μg·hr/mL)**	15.9977	7.5215	Females 52.98% lower AUC_inf_
**t**_1**/2**_ **(hr)**	11.4017	11.0805	Females 2.82% lower t_1/2_
**AUCextra**	79.4791	14.1974	AUCextra 82.14% lower in females
**CL (mg/μg·hr/mL)**	1.2502	22.6591	1712.43% increase in female CL
**Vz**	20.5644	42.5073	106.70% increase in female Vz

**Table 5:** Consolidated Non-Compartmental Pharmacokinetic Summary of Non-Irradiated Male and Female C57BL/6 Mice Following Oral Administration of 40 mg/kg of YK-4-250.

PK Parameter	Male Mice (M)	Female Mice (F)	Sex-Based Variance Analysis (% Difference or Shift)
**C_max_** **(μg/mL)**	1.3994	2.0026	Females exhibit a 43.10% higher C_max_
**T**_**max**_ **(hr)**	1	1	T_max_ are identical
**AUC_last_** **(μg·hr/mL)**	12.5295	18.9944	Females exhibit an 51.60% higher AUC_last_
**AUC**_**inf**_ **(μg·hr/mL)**	13.1428	19.6764	Females 49.71% higher AUC_inf_
**t**_**1/2**_ **(hr)**	5.6830	12.0281	Females 111.65% higher **t**_1_/_2_
**CL (mg/μg·hr/mL)**	3.0435	2.0329	33.21% lower CL in females
**Vz**	24.9531	35.2766	41.37% increased Vz in females

**Table 6:** Consolidated Non-Compartmental Pharmacokinetic Summary of Irradiated Male and Female C57BL/6 Mice Following Oral Administration of 20 mg/kg of YK-4-250.

PK Parameter	Male Mice (M)	Female Mice (F)	Sex-Based Variance Analysis (% Difference or Shift)
**C_max_** **(μg/mL)**	0.7981	0.9110	Females exhibit an 14.15% higher C_max_
**T**_**max**_ **(hr)**	3	1	66.67% decrease in female T_max_
**AUC_last_** **(μg·hr/mL)**	3.9392	6.2306	Females exhibit a 58.17% higher AUC_last_
**AUC**_**inf**_ **(μg·hr/mL)**	4.3724	8.8047	Females have 101.37% higher AUC_inf_
**t**_**1/2**_ **(hr)**	3.1210	7.8141	85.23% increased t_1/2_ in females
**CL (mg/μg·hr/mL)**	4.8040	2.3954	50.137% decreased CL in female
**Vz**	21.6306	27.0048	24.85% increase in female Vz

**Table 7:** Consolidated Non-Compartmental Pharmacokinetic Summary of Irradiated Male and Female C57BL/6 Mice Following Oral Administration of 40 mg/kg of YK-4-250.

PK Parameter	Male Mice (M)	Female Mice (F)	Sex-Based Variance Analysis (% Difference or Shift)
**C_max_** **(μg/mL)**	1.8951	1.6815	Females exhibit an 11.27% lower C_max_
**T**_**max**_ **(hr)**	3	6	100% increase in female T_max_
**AUC_last_** **(μg·hr/mL)**	20.6031	22.0872	Females exhibit a 7.20% higher AUC_last_
**AUC**_**inf**_ **(μg·hr/mL)**	22.0124	22.6795	Females have 3.03% higher AUC_inf_
**t**_**1/2**_ **(hr)**	22.5087	18.7456	Dramatic increased in both sexes; 16.72% decrease in t_1/2_ in females as compared to males
**CL (mg/μg·hr/mL)**	1.8172	1.7637	2.94% decreased CL in female
**Vz**	59.0088	47.6980	19.17% decrease in female Vz

**Table 8:** Consolidated Pharmacokinetic Summary of YK-4-250 in Mic

Experiment	Irradiation, Sex	Dose mg	C_max_ μg/mL	T_max_ hr	AUC_last_ μg.hr/mL	AUC_inf_ μg.hr/mL	AUC Extra-polated %	T_1/2_ hr	MRT hr	CL mg/(μg. hr/mL	Vz apparent	lambda z/hr	Lambda zR^2^
PBI-035-2	No IR, M	20	1.1414	1	3.7628	15.9977	76.4791	11.4017	17.8697	1.2502	20.5644	0.0608	0.0739
PBI-035-3	No IR, M	40	1.3994	1	12.5295	13.1428	4.6662	5.683	8.8574	3.0435	24.9531	0.122	0.9002
PBI-036-2	No IR, F	20	1.2903	1	6.4536	7.5215	14.1974	11.0805	10.4092	22.6591	42.5073	0.0626	0.7850
PBI-036-3	No IR, F	40	2.0026	1	18.9944	19.6764	3.4659	12.0281	10.5252	2.0329	35.2766	0.0576	0.9487
PBI-037-2	IR, M	20	0.7981	3	3.9392	4.3724	9.9065	3.121	5.3498	4.5741	20.5957	0.2221	0.9154
PBI-037-3	IR, M	40	1.8951	3	20.6031	22.0124	6.4025	22.5087	18.9935	1.8172	59.0088	0.0308	0.8346
PBI-038-2	IR, F	20	0.9110	1	6.2366	8.8047	29.1672	7.8141	10.3541	2.2715	25.6077	0.0887	0.5366
PBI-038-3	IR, F	40	1.6815	6	22.0872	22.6795	2.6115	18.7456	15.0849	1.7637	47.698	0.037	0.9828

## Data Availability

All data generated or analyzed during this study are included in this published article and its supplementary information file.
